# Ageing influences detrusor contractions to prostaglandin, angiotensin, histamine and 5-HT (serotonin), independent to the Rho kinase and extracellular calcium pathways

**DOI:** 10.1038/s41598-023-44916-8

**Published:** 2023-10-23

**Authors:** Charlotte Phelps, Russ Chess-Williams, Christian Moro

**Affiliations:** Centre for Urology Research, Faculty of Health Sciences and Medicine, Bond University, Gold Coast, QLD 4226 Australia

**Keywords:** Urology, Bladder

## Abstract

Ageing is associated with deteriorating urinary bladder function and an increasing prevalence of disorders such as underactive bladder. There are suggestions that G protein-coupled receptor (GPCR) second messenger pathways are altered during ageing, rather than the receptor proteins themselves. The aim of this study was to identify age-related variations in GPCR activation systems in urinary bladder smooth muscle (detrusor). Isolated porcine detrusor strips were mounted in organ baths and contractile responses induced by receptor agonists were assessed and compared between juvenile (6 months) and adult (2 years) animals. The effects of drugs disrupting intracellular calcium signalling were also studied. Adult tissue was far more sensitive to stimulation by 5-hydroxytryptamine (42% greater increase than juvenile), prostaglandin-E2 (26% greater increase), and angiotensin-II (39% greater increase), however less sensitive to histamine. Although nifedipine and Y-27632 impacted the contraction to all agonists, there were no significant differences between juvenile and adult detrusor. Impairment of IP3-mediated calcium release by 2-aminoethyl diphenylborinate had no effect on any contractile activity, except for neurokinin-A which inhibited both juvenile and adult detrusor, and prostaglandin-E2 which inhibited juvenile. Carbachol, histamine, 5-hydroxytryptamine, and angiotensin-II were not affected by the application of 2-aminoethyl diphenylborinate. In conclusion, the contractile responses to all the GPCR agonists involved extracellular calcium influx and calcium sensitisation, but for prostaglandin-E2 the dependence on calcium from intracellular sources was greater in the younger animals.

## Introduction

Underactive bladder (UAB) is a prevalent disorder affecting males and females of all ages. Despite significant impacts on quality of life, UAB is frequently misdiagnosed due to the lack of a detailed definition and diagnostic criteria in clinical practice, with the pathophysiology remaining poorly understood^1^. As a result, there are currently no outcome-validated effective therapeutics for the management or prevention of UAB^2^. The front-line pharmaceutical treatment for UAB is parasympathomimetics (muscarinic agonists), which act to directly stimulate muscarinic receptors or inhibit anticholinesterase to increase cholinergic neurotransmission^2^. However, there is a paucity of quality evidence suggesting long-term benefits for this class of medicine^3,4^, and there are a range of adverse effects associated with parasympathomimetic use, such as nausea, vomiting, diarrhea, sweating, and salivation, which lead to low adherence rates^5^.

Diminished urinary bladder function and control is a frequent occurrence among elderly populations^6^. Ageing is associated with various alterations in urinary bladder structure and function, resulting in changes to bladder storage, contraction, emptying, and sensitivity^7,8^. These age-induced alterations contribute to the increasing prevalence of lower urinary tract symptoms (LUTS) with progressing age^6^. In elderly men and women with LUTS, over 45% exhibit an underactive bladder, presenting this as an increasingly prevalent problem that commonly results in urinary retention, incomplete bladder emptying, and other bothersome urinary symptoms, such as nocturia, urgency, hesitancy, and incontinence^9,10^. UAB is often a result of detrusor underactivity, which is a urodynamic diagnosis characterised by a reduced contraction strength or length of prolonged voiding, resulting in incomplete bladder emptying^4^. However, the direct mechanisms underlying UAB are complex to diagnose and manage, and as ageing is a predictor of developing UAB, this highlights the need to further enhance understanding of age-related changes in bladder contraction. Furthermore, persistence and adherence to some pharmaceutical therapies for bladder contractile disorders increase with age, suggesting variations in bladder responses to receptor agonists^11,12^.

The detrusor smooth muscle remains the primary target in the pharmaceutical therapy of bladder contractile disorders^13^. For UAB patients, the primary treatment goal is to enhance the contractile activity of the urinary bladder to increase the strength and duration of contractions to complete bladder emptying^14^. Smooth muscle contraction is stimulated by an increase in intracellular calcium (Ca^2+^) via an influx of extracellular Ca^2+^ through voltage-gated Ca^2+^ channels or triggering its release from intracellular sarcoplasmic reticulum stores. The influx of intracellular Ca^2+^ activates myosin light chain kinase, which phosphorylates myosin light chain and promotes the interaction of myosin with actin and subsequent contraction. G protein-coupled receptor (GPCR) activation is a primary stimulus for increases in intracellular Ca^2+^ concentration^15^. Agonists binding to GPCRs activate voltage-dependent Ca^2+^ channels to increase intracellular Ca^2+^, which increases the strength and force of contraction with the subsequent influx of extracellular Ca^2+^^16^. Further, G_q/11_-coupled receptors activate phospholipase C with the subsequent formation of inositol trisphosphate (IP3) and diacylglycerol to release Ca^2+^ from intracellular stores^17^. Smooth muscle contraction is also initiated by a Ca^2+^-independent pathway via activation of Rho kinase, achieved by inhibiting the counteracting enzyme myosin light chain phosphatase leading to sustained contraction^18^.

Pharmaceutical treatments for UAB target the G_q/11_-coupled M3 muscarinic receptors within the urinary bladder wall^2^. However, due to low adherence rates from lower-than-expected outcomes and side effects associated with parasympathomimetic use, there has been increasing interest in novel targets in the urinary bladder for future treatment development^2^. Of particular interest are the G_q/11_-coupled receptors, which mediate contractions in the bladder. Recent research has highlighted the role of histamine, 5-hydroxytryptamine (5-HT, serotonin), neurokinin-A (NKA), prostaglandin E2 (PGE2), and angiotensin-II (ATII) receptors within the various layers of the bladder wall contributing to contraction^15^.

A range of G_q/11_ receptors have been observed to mediate contraction in healthy urinary bladders, with responses influenced by Ca^2+^ influx from extracellular sources^15^, as well as Rho kinase Ca^2+^-sensitisation pathways^17^. Studies have also shown age-related alterations in GPCR-mediated contractile responses in the urinary bladder, for example, muscarinic^19^, histamine^20^, 5-HT^21^, and prostaglandin^22^. Inhibited GPCR-induced contractions may be related to decreased receptor density or down-regulation by ageing^23^, or may be caused by alterations downstream of smooth muscle receptors such as depressed intracellular pathways involved in contraction^24^. It has been suggested that it is not the contractile or cytoskeleton proteins that are affected by ageing^25^, but it is more likely due to alterations in the intracellular mechanisms involving secondary messengers that may affect the response of the bladder to agonists^24^. Ageing has been shown to increase the dependence of muscarinic receptor-mediated detrusor smooth muscle contractions on extracellular Ca^2+^ influx^26^, and alter Ca^2+^-dependent and -independent contraction pathways^27^. There are also suggestions that Rho kinase activity may be altered with ageing, affecting carbachol-induced contractions of the detrusor smooth muscle^28^. However, age-related alterations to these pathways and their influence on smooth muscle contraction have not been well-defined across the various other receptor systems.

Porcine tissue has been increasingly used as a research model for healthy human tissue. Although there is currently no validated model of underactive bladder that replicates human presentations, assessing the influence of development between juvenile and adult porcine bladders is likely to provide insights into how age can influence receptor activation and contractility in the urinary bladder. This study aimed to assess the influence of prominent G_q/11_ receptor signalling pathways, chosen for their ability to induce strong contractions of detrusor smooth muscle, between juvenile and adult urinary bladders.

## Results

### GPCR agonist stimulation of detrusor contractile activity

**Figure 1:**
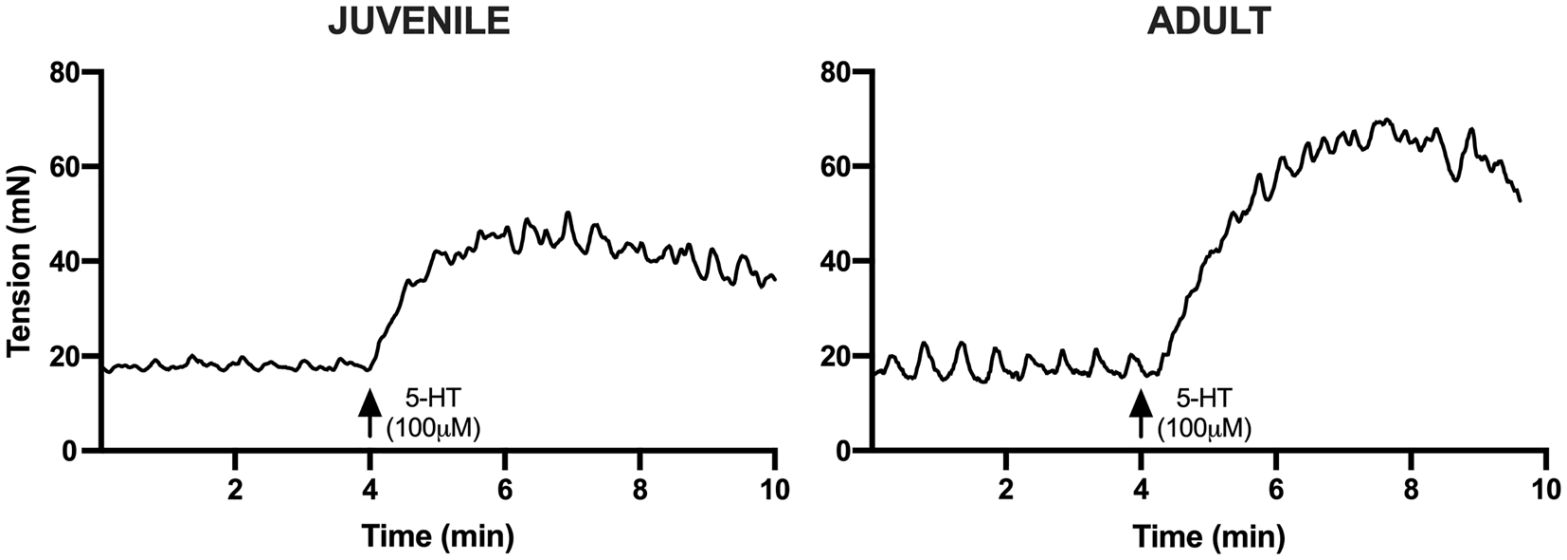
Representative experimental trace comparing juvenile (left) and adult (right) detrusor tension response to 5-HT (100 μM). Note the significantly greater response in the adult detrusor compared to the juvenile detrusor.

The mean ± SEM baseline tension in the absence of any stimulation for juvenile detrusor tissues was 44.57 ± 0.92 mN/g (*n* = 144) and for adult tissues was 38.25 ± 0.93 mN/g (*n* = 144). Table 1 presents the change in baseline tension for juvenile and adult detrusor smooth muscle in response to activation of GPCRs with the agonists carbachol (1 μM), histamine (100 μM), 5-HT (100 μM), NKA (300 nM), PGE2 (10 μM), and ATII (100 nM). Juvenile tissues showed a significantly greater tension response to histamine (100 μM) compared to adult tissues (*n* = 22, *p* = 0.04). In detrusor preparations from adult pigs, 5-HT (100 μM, Fig. 1), PGE2 (10 μM), and ATII (100 nM) elicited significantly greater tension responses when compared to tissues from juvenile animals (Table 1). There was no significant difference between juvenile and adult responses to muscarinic and NKA receptor activation.

**Table 1:** Comparison of responses to GPCR activation between juvenile and adult detrusor tissues.

Agonist (concentration)	Δ Tension (mN)		*n*	*p*-value
	Juvenile	Adult		
Carbachol (1 μM)	112.95 ± 12.75	128.18 ± 17.39	24	0.48
**Histamine (100 μM)**	**15.35 ± 2.94**	**8.63 ± 1.27**	**22**	**0.04**
**5-HT (100 μM)**	**26.09 ± 4.53**	**44.82 ± 7.54**	**22**	**0.04**
NKA (300 nM)	35.99 ± 6.65	52.32 ± 8.50	24	0.14
**PGE2 (10 μM)**	**22.93 ± 1.81**	**31.15 ± 3.21**	**24**	**0.03**
**ATII (100 nM)**	**6.40 ± 1.25**	**10.52 ± 1.50**	**24**	**0.04**

Data reported as mean ± SEM.

Bold font indicates statistical significance (*p* < 0.05, unpaired Student’s two-tailed *t*-test).

### Extracellular Ca^2+^ for GPCR activation of juvenile and adult tissues

**Figure 2:**
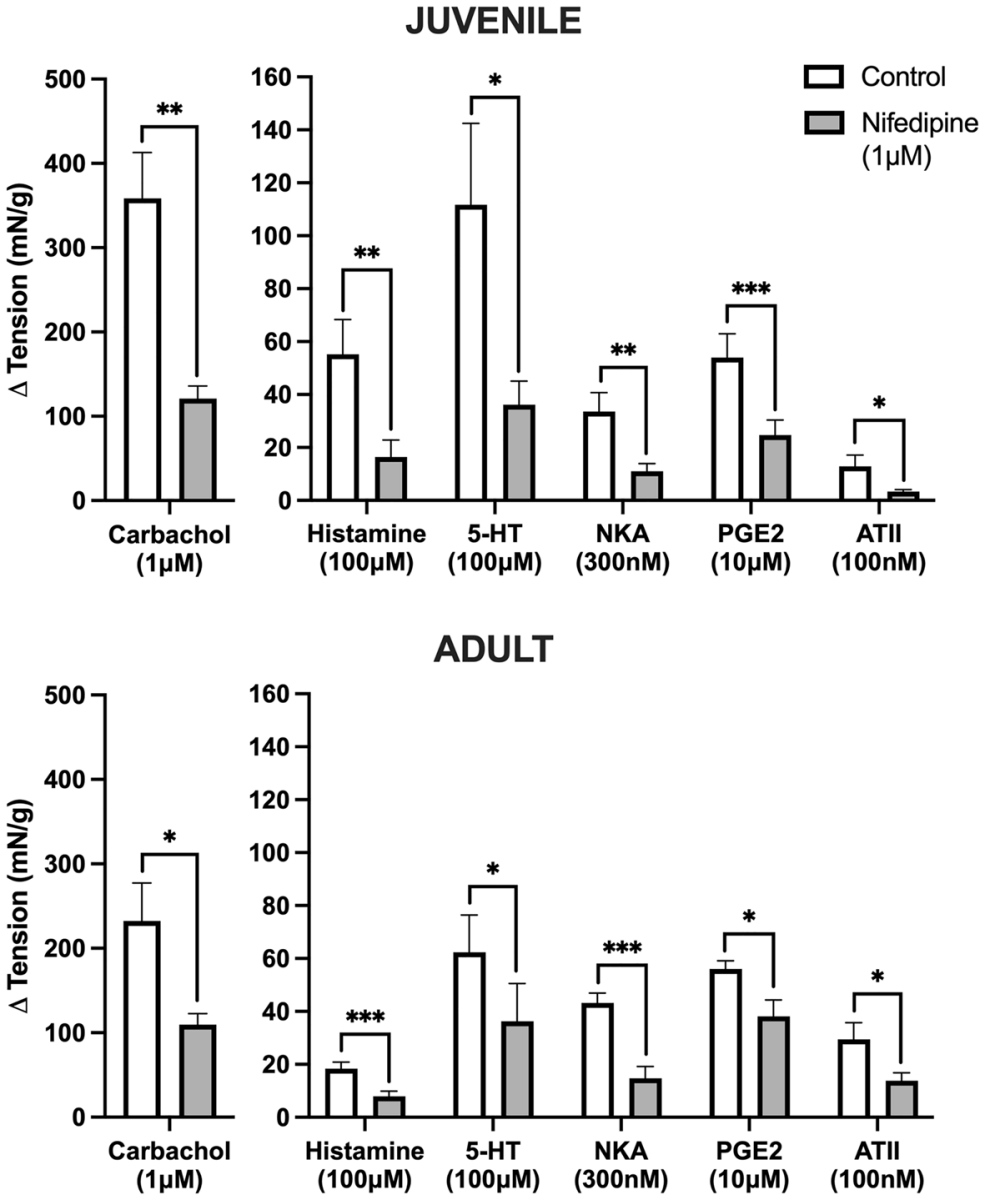
The influence of 1 μM nifedipine on GPCR-mediated contractile responses in juvenile (upper chart) versus adult (lower chart) detrusor smooth muscle (*n* = 8 for all). *p < 0.05, **p < 0.01, ***p < 0.001 (paired Student’s two-tailed *t*-test). Data reported as mean ± SEM. As carbachol induced a stronger contraction than the other receptors, the y-axis for this data set was expanded.

In both juvenile and adult detrusor smooth muscle, blocking extracellular Ca^2+^ influx with nifedipine (1 μM) reduced all agonist responses (Fig. 2). In juvenile detrusor, all responses were significantly inhibited by nifedipine (*p* < 0.05 for all, paired Student’s two-tailed *t*-test), for carbachol (Δ 238.59 ± 60.73 mN/g, 1 μM, *n* = 8), histamine (Δ 38.82 ± 10.66 mN/g, 100 μM, *n* = 8), 5-HT (Δ 75.56 ± 22.83 mN/g. 100 μM, *n* = 8), NKA (Δ 22.54 ± 5.91 mN/g, 300 nM, *n* = 8), PGE2 (Δ 29.30 ± 4.95 mN/g, 10 μM, *n* = 8), and ATII (Δ 9.56 ± 3.73 mN/g, 100 nM, *n* = 8). The depressions were also consistent with the adult detrusor, where application of nifedipine (1 μM, *p* < 0.05 for all) impacted responses to carbachol (Δ 122.80 ± 39.70 mN/g, 1 μM, *n* = 8), histamine (Δ 10.49 ± 1.48 mN/g, 100 μM, *n* = 8), 5-HT (Δ 26.01 ± 9.65 mN/g, 100 μM, *n* = 8), NKA (Δ 28.49 ± 3.57 mN/g, 300 nM, *n* = 8), PGE2 (Δ 17.87 ± 5.92 mN/g, 10 μM, *n* = 8), and ATII (Δ 15.70 ± 6.27 mN/g, 100 nM, *n* = 8).

#### Comparison between juvenile and adult

Responses to each agonist for both age groups were significantly depressed after the application of nifedipine. When comparing the magnitude of depression between juvenile and adult tissues, the impact of nifedipine was similar between age groups, with no significant differences for any of the agonists (*p* = NSD for all, unpaired Student’s two-tailed *t*-test).

### Intracellular Ca^2+^ for GPCR activation of juvenile and adult tissues

**Figure 3:**
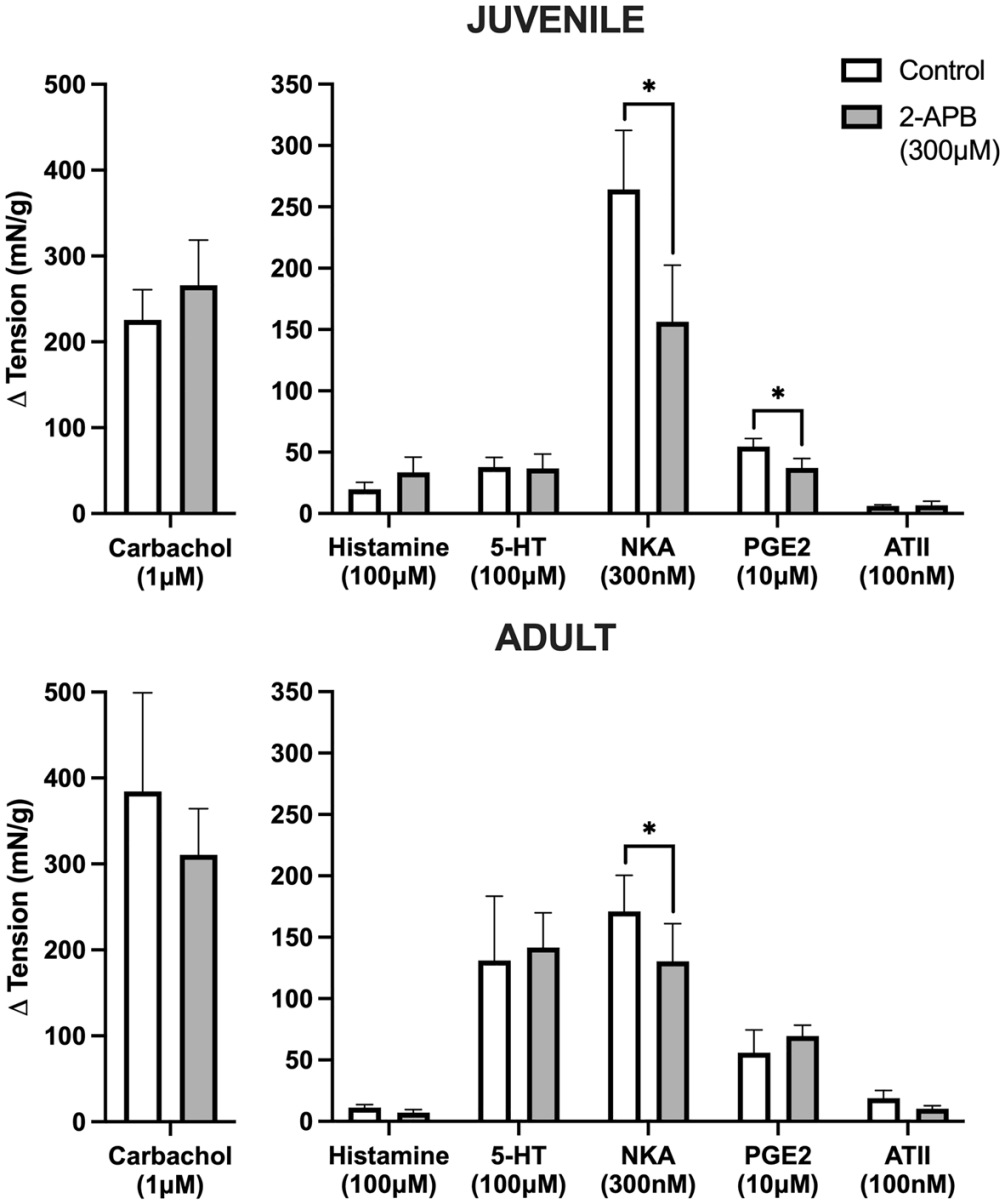
The influence of 300 μM 2-APB on GPCR-mediated contractile activity in juvenile (top) versus adult (bottom) detrusor smooth muscle (*n* = 8 for all). *p > 0.05 (paired Student’s two-tailed *t*-test). Data reported as mean ± SEM.

After application of an inhibitor of IP3-induced Ca^2+^ release, 2-APB (300 μM), there was no significant difference in the change in baseline tension for responses to carbachol (1 μM, *n* = 8), histamine (100 μM, *n* = 8), 5-HT (100 μM, *n* = 8), and ATII (100 nM, *n* = 8) for both juvenile and adult detrusor smooth muscle (Fig. 3). GPCR-mediated contraction in the presence of 2-APB compared to controls was reduced in both juvenile and adult detrusor tissues in response to NKA (300 nM) by 38% (*n* = 8, *p* = 0.04) and 31% (*n* = 8, *p* = 0.004) respectively, with no significant difference between the two groups. However, after the application of 2-APB, a difference *was* recorded between juvenile and adult responses to PGE2 (10 μM, *p* = 0.03, unpaired Student’s two-tailed *t*-test). While the response to PGE2 activation was inhibited in juvenile samples by 35% (*n* = 8, *p* = 0.02), there was no inhibition in aged samples (Fig. 3).

### Rho kinase pathway for GPCR activation of juvenile and adult tissues

**Figure 4:**
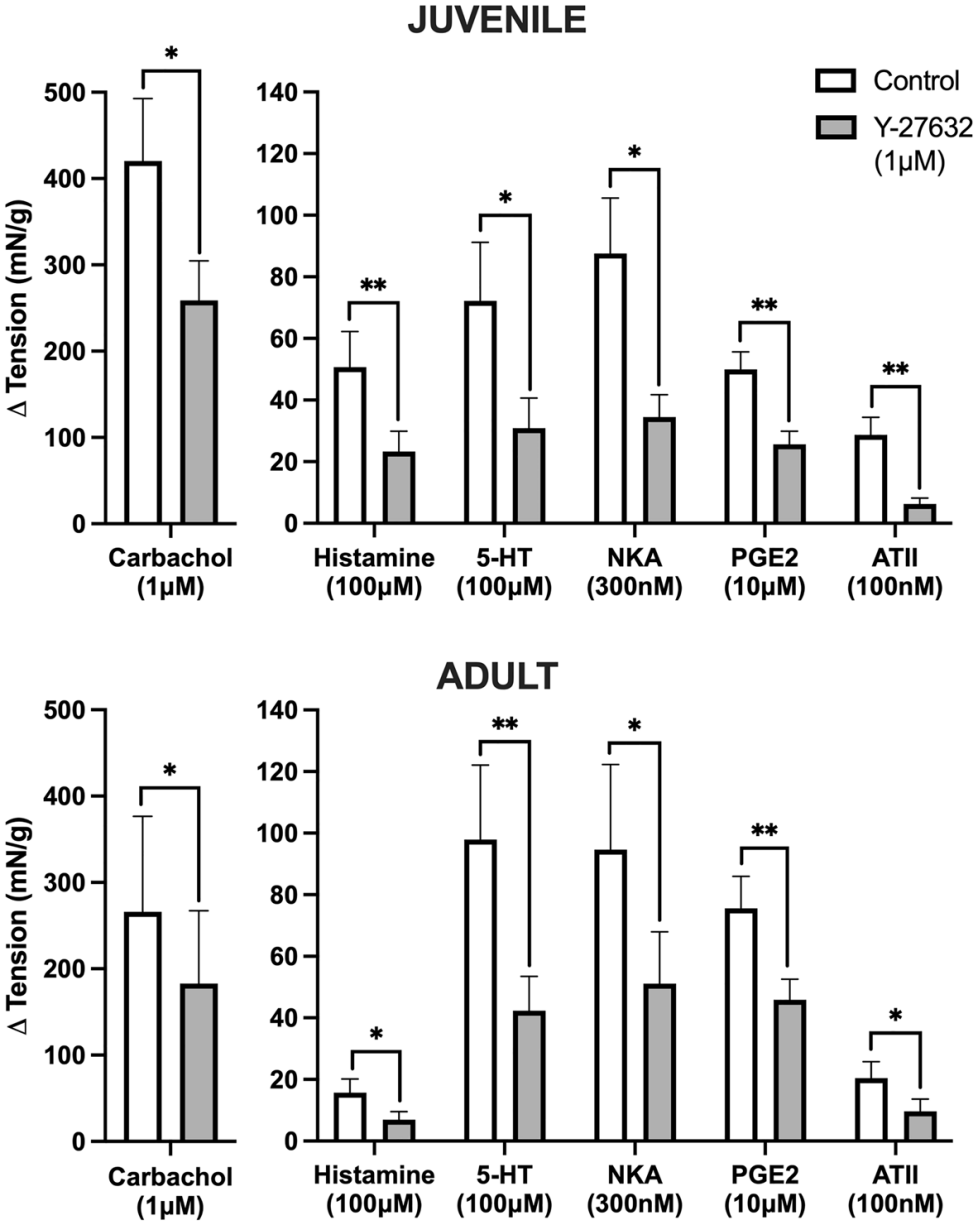
The influence of 1 μM Y-27632 on contractile activity in juvenile (top) versus adult (bottom) detrusor smooth muscle in response to agonists carbachol (*n* = 8), histamine (*n* = 6), 5-HT (*n* = 6), NKA (*n* = 8), PGE2 (*n* = 8), and ATII (*n* = 8). *p > 0.05, **p > 0.01 (paired Student’s two-tailed *t*-test). Data reported as mean ± SEM.

After activation of the six GPCRs with agonists, in the presence of the Rho kinase inhibitor, Y-27632 (1 μM), increases in the baseline tension of detrusor smooth muscle tissues for both juvenile and adult samples was significantly reduced compared to controls (*p* < 0.05 for all, paired Student’s two-tailed *t*-test, Fig. 4). Inhibition of GPCR-mediated contractions by Y-27632 for juvenile and adult detrusor samples, respectively, was: 1 μM carbachol by 42% and 34% (*n* = 8); 100 μM histamine by 66% and 61% (n = 6); 100 μM 5-HT by 60% and 52% (*n* = 6); 300 nM NKA by 51% and 52% (*n* = 8); 10 μM PGE2 by 47% and 43% (*n* = 8); and 100 nM ATII by 78% and 54% (*n* = 8). When comparing the difference between juvenile and adult responses to the inhibition of Rho kinase, Y-27632 was equally as effective at inhibiting contractile activity between the two age groups, with no significant differences (unpaired Student’s two-tailed *t*-test) between the magnitude of responses to any of the agonists.

## Discussion

As the prevalence of underactive bladder increases with age, this study sought to investigate different time points to see how receptor-mediated contractile activity of the urinary bladder differs. Using porcine detrusor smooth muscle, various G protein-coupled receptor systems and their downstream signalling pathways that contribute to detrusor smooth muscle contractions were explored. The findings from this research highlight the varying responses to key mediators of contraction between juvenile and adult urinary bladder smooth muscle. Whilst the alterations do not appear to be related to G_q/11_-coupled second messenger pathways, particularly extracellular Ca^2+^ or Rho kinase, there were differences in the sensitivity to intracellular Ca^2+^.

The activation of the muscarinic, histamine, 5-HT, NKA, PGE2, or ATII receptors increased the force of contraction in both juvenile and adult urinary bladder detrusor smooth muscle. Muscarinic receptors, in particular the M3 muscarinic receptor remain the most widely studied receptor in the detrusor^29^ and urothelium/lamina propria^30^, as they are primarily responsible for mediating contractions through cholinergic activation, and therefore remain the main target for treating bladder contractile disorders^31^. However, due to the side effects and lower-than-expected treatment outcomes associated with muscarinic receptor therapeutics^2^, there is a growing interest in additional receptor systems in the urinary bladder that may present novel targets for future pharmaceutical development. Of note, the H1 histamine^32^, 5-HT_2A_^33^, neurokinin-2^34^, EP1 prostaglandin E2^35^, and AT_1_ angiotensin II^15^ receptors are known to cause contractions in the urinary bladder, and therapeutics that target these G_q/11_-coupled receptors could be effective in the treatment of bladder contractile disorders, such as underactive bladder.

Across the GPCRs explored, developmental differences were identified in detrusor contractile activity in response to some of the agonists. Contractile responses to histamine in the adult detrusor were significantly smaller when compared to contractions observed in juveniles, which is consistent with findings by Stromberga et al.^20^. This could be explained by reduced compliance in the adult detrusor as a result of a greater deposition of collagen in the aged bladder^36,37^. In contrast, the urinary bladder of adult detrusor responded to a variety of agonists, including 5-HT, PGE2, and ATII, to a higher degree than the juvenile detrusor. It is known that these agonists are involved in bladder contraction, and the finding that the detrusor contractile response is more sensitive with age may indicate involvement in bladder dysfunction.

Beyond receptor activation, intracellular signalling mechanisms contributing to contraction could also be accessible targets for pharmaceutical development^38^, and these may also be altered with age. Extracellular Ca^2+^, intracellular Ca^2+^, and Rho kinase are important components of the G_q/11_-coupled contraction pathway^39^, and alterations to the availability of these molecules for contraction may contribute to the development of bladder contraction disorders.

The contribution of extracellular Ca^2+^ and Rho kinase for contractions was consistent across both juvenile and adult detrusor, indicating that extracellular Ca^2+^ entry into the tissue via voltage-gated Ca^2+^ channels and Ca^2+^ sensitisation via the Rho kinase pathway is unlikely to be impaired with age for various G_q/11_ receptors known to cause contraction. The influence of Ca^2+^ from intracellular sources for agonist-induced contractions of tissues was minimal, and this was similar for the two age groups. This highlights that there is a stronger dependence on extracellular Ca^2+^ and Rho kinase for mediating muscarinic, histamine, 5-HT, and ATII detrusor contractions. Impairment of IP3-mediated Ca^2+^ release with 2-APB inhibited contractions induced by NKA for both juvenile and adult detrusor samples and juvenile samples inhibited in response to PGE2. These findings are unique to these receptors in the detrusor, as the influence of intracellular Ca^2+^ release was found to be minimal for neurokinin and prostaglandin receptors in the urothelium and lamina propria^17^. The finding that there was decreasing sensitivity to intracellular Ca^2+^ in response to PGE2-mediated contractions during ageing indicates an age-related difference in the G_q/11_ second messenger pathway. The EP1 prostaglandin E2 receptor may be of particular interest in future studies, as the magnitude of inhibition was significantly greater in juvenile detrusor than adult detrusor. This may be supported by findings that urinary bladder PGE2 levels are negatively correlated with age^40,41^, and as such, further investigations focussing on the receptor expressions could assist. One hypothesis for this age-related difference is that the juvenile detrusor may be more dependent on IP3-mediated Ca^2+^ release for PGE2-induced contractions, whereas activation of the prostaglandin receptors in the adult detrusor relies on an increase in extracellular Ca^2+^, which may activate ryanodine receptors to induce intracellular Ca^2+^ release^42^.

## Limitations and future directions

Although a viable and well-validated model, porcine tissue may not directly correlate to human function, and experiments that replicate this study on human tissue would be of interest. It is also unclear which age would be equivalent to a 2-year-old sow. These animals are past their reproductive prime, and often referred to as the older pigs. However, it would likely not be correct to classify these animals as “elderly”. As such, the terms juvenile and adult were used throughout, and future studies could investigate different age groups. Further, finding the molecular mechanisms involved in the differences observed would present an interesting direction for future investigations, as well as comparing different animal models. Lastly, future studies could explore age-related alterations in the signalling pathways associated with GPCR-mediated contractions across the various tissue layers in the urinary bladder, such as the urothelium and lamina propria.

The increased contraction to 5-HT, PGE2, and ATII, and decreased sensitivity to histamine in older tissue, may provide insights into potential systems that could contribute to dysfunction in the contractility of the urinary bladder. In most cases, these observed alterations do not appear to be related to G_q/11_-coupled second messenger pathways that involve extracellular Ca^2+^ or Rho kinase, highlighting a potentially important age-related alteration. Alternatively, the decreased sensitivity to intracellular Ca^2+^ in response to PGE2-mediated contractions in aged tissues, compared to juvenile samples may also warrant further investigation. Overall, juvenile and adult bladders demonstrate clear differences in their ability to contract in response to agonist stimulation, suggesting additional mechanisms that may contribute to bladder contractile dysfunction.

## Methods

### Tissue collection

Porcine urinary bladders from Large White-Landrace-Duroc cross-bred pigs (*Suf scrofa domestica*) were obtained from the local abattoir after slaughter for the routine commercial provision of food. Juvenile samples were taken from prepubescent pigs aged 6 months old at 80 kg live weight, and adult samples from sow pigs aged 2–3 years old at 200 kg live weight. After collection from the abattoir, tissues were transported in a portable cooler in cold Krebs–Henseleit bicarbonate solution (Krebs, composition in mM: NaCl 118.4; NaHCO_3_ 24.9; d-glucose 11.7; KCl 4.6; MgSO_4_ 2.41; CaCl_2_ 1.9; and KH_2_PO_4_ 1.18) maintained at 4 °C to the University research facilities and used within three hours of the animal’s slaughter.

### Tissue preparation

Strips measuring 2.0 cm × 0.5 cm were dissected longitudinally from the urinary bladder dome. The detrusor smooth muscle was carefully isolated from the urothelium, lamina propria, and serosal tissue layers using fine scissors^43^. Adjacent paired tissue strips were mounted and suspended in 10 mL organ baths (Labglass, Brisbane, Australia) containing Krebs bicarbonate solution at a constant temperature of 37 °C and gassed with carbogen (95% oxygen and 5% carbon dioxide). After mounting, tissues were equilibrated for 15 min, and each bath was washed through with warmed Krebs a total of three times prior to the addition of any pharmaceuticals. The tension placed on the tissues was manually adjusted to 20mN using a moveable isometric force transducer (MCT050/D, ADInstruments, Castle Hill, Australia) with a fine adjustment level. At the conclusion of each experiment, tissue strips were dried and weighed, and the mean net weight of porcine detrusor tissue for juvenile samples was 0.39 ± 0.01 g (*n* = 288) and for adult samples was 0.52 ± 0.01 g (*n* = 288), with significant differences between specimens (*p* < 0.01, unpaired Student’s two-tailed *t*-test). As such, data has been presented as millinewton force per gram tissue weight (mN/g) to accommodate for the different weights. The number of tissues (*n*) is quoted from paired tissue strips, therefore the number of animals (*N*) used can be calculated by using *n* ÷ 2.

### Measurements and data collection

After equilibration, selective inhibitors of extracellular Ca^2+^ influx, intracellular Ca^2+^ release, or Rho kinase were added separately to tissues for 30 min and contractile responses were assessed by performing single-dose agonist studies. After equilibration, a single dose of a selective GPCR agonist was added to both the control and experimental tissues after equilibration. Baseline tension was measured with an isometric force transducer and recorded on a Powerlab system using Labchart v7 software (ADInstruments). Changes in baseline tension were expressed as millinewton force per gram tissue weight (mN/g).

### Pharmaceutical agents

Carbamylcholine chloride (carbachol), histamine dihydrochloride (histamine), and 2-aminoethyl diphenylborinate (2-APB) were obtained from Sigma-Aldrich (Missouri, US). Neurokinin-A (NKA) and nifedipine were from Tocris Bioscience (Bristol, UK). Serotonin hydrochloride (5-HT) was from Toronto Research Centre (Toronto, CA). Angiotensin II (ATII) and prostaglandin E2 (PGE2) were obtained from Cayman Chemicals (Michigan, US), and Y-27632 hydrochloride (Y-27632) from AdooQ BioScience (Irvine, CA). PGE2 and nifedipine were dissolved in 100% ethanol, 2-APB was dissolved in dimethyl sulfate, and all other pharmaceutical agents were soluble in distilled water. Nifedipine was stored in the dark until the final application in the organ bath to ensure no adverse light impacts and experiments were concluded within a 30-min period. Concentrations chosen for the agonists and antagonists were selected based on their selectivity at each receptor to produce a submaximal contraction (~ 80%) and consistent with concentrations used in previous studies using porcine tissue.

### Statistical analysis

Statistical analysis of data was undertaken using GraphPad Prism version 10 (San Diego, CA). Results were presented as mean ± standard error of the mean (SEM). A paired Student’s two-tailed *t*-test was used to compare tissue responses to their direct controls, and an unpaired Student’s two-tailed *t*-test was applied to make comparisons between juvenile and adult groups. In all cases, *p* < 0.05 was considered statistically significant.

### Ethics

As no animals were bred, harmed, culled, interfered, or interacted with as part of this research project, animal ethics approval was not required. Experimental protocols remained in accordance with the Australian Code of Practice for the Care and Use of Animals for Scientific Purposes^44^.

## Data Availability

The datasets generated during and/or analysed during the current study are available from the corresponding author on reasonable request.
